# P-533. CD4 T-cell, CD4/CD8 Ratio Improvement and a General Reduction in Inflammatory Biomarkers with Low-Level Viremia (LLV) up to Week 192 with Fostemsavir (FTR)-Based Regimens in Individuals with Multidrug-Resistant (MDR) HIV-1

**DOI:** 10.1093/ofid/ofae631.732

**Published:** 2025-01-29

**Authors:** Vincenzo Spagnuolo, Natalia Gregori, Iacopo Marcon, Fangfang Du, Bo Li, Marcia Wang, Alftan Dyson, Manyu Prakash, Andrew Clark

**Affiliations:** Vita-Salute San Raffaele University; Unit of Infectious Diseases, IRCCS, San Raffaele Scientific Institute, Milan, Emilia-Romagna, Italy; ViiV Healthcare, Verona, Veneto, Italy; ViiV Healthcare, Verona, Veneto, Italy; Temple University, Chesterbrook, Pennsylvania; GSK, Collegeville, Pennsylvania; GlaxoSmithKline, Upper Providence, Pennsylvania; ViiV Healthcare, Verona, Veneto, Italy; ViiV Healthcare, Brentford, UK, Brentford, England, United Kingdom; ViiV Healthcare, Brentford, UK, Brentford, England, United Kingdom

## Abstract

**Background:**

Persistent LLV (40-1000 c/mL) is associated with virologic failure, drug resistance and increased risk of inflammation and may impact morbidity and mortality. FTR (prodrug of the first-in-class attachment inhibitor temsavir) is indicated with other antiretrovirals (ARVs) for heavily treatment-experienced individuals with MDR HIV-1 unable to construct suppressive regimens. BRIGHTE participants (pts) did not have to stop FTR due to LLV. We describe outcomes and inflammatory biomarkers through Week 192 in pts with LLV (< 40, 40-400, 400-1000 and > 1000 c/mL) on FTR-based regimens from the Randomized Cohort (RC) in the BRIGHTE study.

**Methods:**

BRIGHTE was a phase 3 study (N=371; RC, n=272; Non-randomized Cohort, n=99) in adults failing their current ARV regimen (HIV-1 RNA > 400 c/mL) with ≤ 2 fully active approved ARVs. Pts with 1 or 2 active ARVs entered the RC and received open-label FTR + optimized background therapy after an 8-day blinded placebo-controlled period. Virologic and immunologic responses were analyzed by baseline (BL) demographics and disease characteristics.

**Results:**

At BL in the RC, 89% of pts had CD4 T-cell count < 350 cells/mm^3^, with 5% between 350 and < 500 cells/mm^3^ and 6% ≥ 500 cells/mm^3^. Mean CD4 T-cell increase observed in the < 40 c/mL (n=142) group was 331 cells/mm^3^, 40-400 c/mL (n=25) was 263 cells/mm^3^, 400-1000 c/mL (n=2) was 218 cells/mm^3^, and in pts with > 1000 c/mL (n=10) was 107 cells/mm^3^. Similar mean increase in CD4/CD8 ratio was observed in pts with LLV; ratio in the < 40 c/mL (n= 142) group was 0.38, 40-400 c/mL (n=25) was 0.31, 400-1000 c/mL (n=2) was 0.32, and in pts with > 1000 c/mL (n=10) was 0.09. Biomarkers showed a mean general reduction; < 40 c/mL (sCD14: −371 µg/L, n=133; sCD163: −134 µg/L, n=40; D-dimer: 0.16 mg/L, n=139), 40-400 c/mL (sCD14: −428 µg/L, n=21; sCD163: −80 µg/L, n=5; D-dimer: −0.14 mg/L, n=22), 400-1000 c/mL (sCD14: 1174 µg/L, n=2; sCD163: no data; D-dimer: 0.04 mg/L, n=2), > 1000 c/mL (sCD14: 271 µg/L, n=10; sCD163: 107 µg/L, n=3; D-dimer: 0.08 mg/L, n=10).

**Conclusion:**

In pts with LLV, there is a persistent increase in CD4 T-cell count and improvement in CD4/CD8 ratio, with a general reduction in inflammatory markers up to Week 192. Results highlight the value of FTR-based regimens for sustained improvement where there is incomplete virologic suppression.

**Disclosures:**

**Vincenzo Spagnuolo, MD**, Gilead Sciences: Advisor/Consultant|Gilead Sciences: Honoraria|MSD: Honoraria|ViiV Healthcare: Honoraria **Natalia Gregori, MD**, GSK: Stocks/Bonds (Public Company)|ViiV Healthcare: employee **Iacopo Marcon, PhD**, GSK: Stocks/Bonds (Public Company)|ViiV Healthcare: employee **Fangfang Du, MS in Statistics**, GSK: Employee|GSK: Stocks/Bonds (Public Company) **Bo Li, PhD**, GSK: Employee **Marcia Wang, PhD**, GSK: Employee|GSK: Stocks/Bonds (Public Company) **Alftan Dyson, PharmD**, GSK: Stocks/Bonds (Public Company)|ViiV Healthcare: Employee **Manyu Prakash, PhD**, GSK: Stocks/Bonds (Public Company)|ViiV Healthcare: employee **Andrew Clark, MD**, GSK: Stocks/Bonds (Public Company)|ViiV Healthcare: employee

